# Autophagy in Plant: A New Orchestrator in the Regulation of the Phytohormones Homeostasis

**DOI:** 10.3390/ijms20122900

**Published:** 2019-06-14

**Authors:** Wentao Gou, Xi Li, Shaoying Guo, Yunfeng Liu, Faqiang Li, Qingjun Xie

**Affiliations:** 1 State Key Laboratory for Conservation and Utilization of Subtropical Agro-Bioresources, Guangdong Provincial Key Laboratory of Plant Molecular Breeding, South China Agricultural University, Guangzhou 510642, China; gwtscau@163.com (W.G.); 18826231837@163.com (X.L.); syguo6688@163.com (S.G.); 2 State Key Laboratory for Conservation and Utilization of Subtropical Agro-Bioresources, College of Life Sciences and Technology, Guangxi University, Nanning 530004, China; yunfengliu_bio@126.com; 3 College of Life Sciences, South China Agricultural University, Guangzhou 510642, China; fqli@scau.edu.cn

**Keywords:** autophagy, phytohormones, crosstalk, stress response, plant growth and development

## Abstract

Autophagy is a highly evolutionarily-conserved catabolic process facilitating the development and survival of organisms which have undergone favorable and/or stressful conditions, in particular the plant. Accumulating evidence has implicated that autophagy is involved in growth and development, as well as responses to various stresses in plant. Similarly, phytohormones also play a pivotal role in the response to various stresses in addition to the plant growth and development. However, the relationship between autophagy and phytohormones still remains poorly understood. Here, we review advances in the crosstalk between them upon various environmental stimuli. We also discuss how autophagy coordinates the phytohormones to regulate plant growth and development. We propose that unraveling the regulatory role(s) of autophagy in modulating the homeostasis of phytohormones would benefit crop breeding and improvement under variable environments, in particular under suboptimal conditions.

## 1. Introduction

Unlike animals, plants are unable to run away from variable and complicated environments, especially stressful conditions. To overcome and/or survive, plants have evolved a sophisticated “self-eating” mechanism similar to animals and other organisms, which is generally referred to as autophagy. Such a biological process is highly conserved among various species, conducting the turnover of biomolecules and unneeded components in bulk. However, a low constitutive level of autophagy in plants is essential for nutrient recycling and metabolic homeostasis. Thus far, the regulatory mechanism(s) of autophagy and the scores of components involved have been well documented, further indicating its importance to the robust crop productivity and yield, especially under suboptimal conditions [1].

The phenomenon and word “autophagy” was first discovered and announced by Christian de Duve with electron microscopy studies in the middle of the 20th century [2]. Because of the lack of effective and systemic approaches, it was thought to be an endocytosis process until the 1990s. The first autophagy-related gene, *Autophagy 1* (*APG1*, and referred to as *ATG1* later), was identified by Yoshinori Ohsumi from a yeast screening. Subsequently, another 14 core *ATGs* were identified and the working model of the autophagic system was characterized [3,4]. Due to his outstanding contributions, Yoshinori Ohsumi won the Nobel Prize in Physiology or Medicine in 2016.

Phytohormones have been evident in the comprehensive housekeeping of plant growth and development. There is a fairly clear picture of phytohormones’ metabolism, transport, perception, and signaling transduction. Large-scale researches have also revealed their roles upon multiple stress responses, including abiotic and biotic stresses, in addition to growth and development [5,6,7,8]. As an example, brassinosteroid (BR) modulates multiple agronomic traits such as plant architecture and grain yield in rice, as well as resistance to a broad range of diseases and various abiotic stresses (salinity stress etc.) [9]. On the other hand, autophagy is also known to be involved in the regulation of plant development and growth in addition to stresses. For example, disruption of *ATG* genes results in pollen defect, leaf senescence and nitrogen use efficiency (NUE) in crop [10,11,12,13,14], whereas enhanced autophagy leads to high tolerance to stresses, increased productivity and yield [15,16,17]. Though both autophagy and phytohormones play broad roles in quiet similar traits/phenotypes, whether there is a crosstalk between them is still poorly understood. The present review is an attempt to highlight the potential connection(s) between them with particular emphasis on the stress regulation.

## 2. Molecular Route of Autophagy Machinery

**Figure 1:**
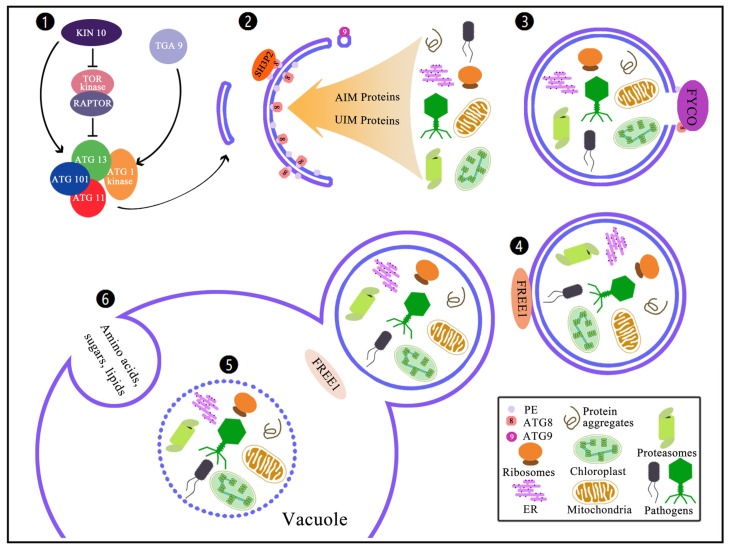
The molecular route of autophagy. (**1**) Induction of macroautophagy is regulated upon favorable and unfavorable conditions: The TOR and RAPTOR kinase complex represses the ATG13-ATG1-ATG101-ATG11 complex to regulate the macroautophagy induction negatively, whereas the KIN10 suppresses the TOR-RAPTOR activity in parallel with the positive role of TGA9 to modulate autophagy induction. (**2**) ATG9 regulates the delivery of lipids to the developing phagophore, while the SH3P2 interacts with ATG8 to decorate autophagosomes, and the AIM or UIM proteins facilitate the transportation of damaged organelles and invasive pathogens to the autophagosomes. (**3**) With the help of FYVE and coiled-coil domain-containing (FYCO) proteins, the autophagosome is tethered to the microtubule transport machinery. (**4**) Fusion of the autophagosomes with the tonoplast is mediated by FYVE-DOMAIN PROTEIN REQUIRED FOR ENDOSOMAL SORTING 1 (FREE1) and other proteins, and then releases autophagic bodies into the vacuole. (**5**) The autophagic bodies are subsequently degraded by vacuolar hydrolases. (**6**) The microautophagy is preceded by invagination of the tonoplast to engulf portions of the cytosolic constituents directly into autophagic bodies within the vacuole, like amino acids, sugars and lipids. Both pathways are dedicated to a cytoplasm-to-vacuole route and eventually lead to the storage and/or recycling of materials.

Autophagy is an evolutionarily-conserved intracellular degradation mechanism participating in multiple biological processes [18]. Distinct from the other degradation systems (such as the ubiquitin-proteasome system), autophagy was characterized by its capability to break down almost all substrates and/or aggregates in cells, including unneeded biomolecules, dysfunctional organelles and even invasive microorganisms [19]. So far, three different types of autophagy have been identified in plant kingdoms: microautophagy, macroautophagy and mega-autophagy [1,20]. All of their molecular routes can be simplified as a cytoplasm-to-vacuole route (Figure 1). In respect to the microautophagy, cytoplasmic congregates are accumulated on the surface of the vacuole and then trapped by tonoplast directly. Subsequently, the vacuole membrane is split to release autophagy, which are intravesicular vesicles containing cytoplasmic components. In contrast, in terms of the macrophage phagocytosis, cargo is captured in the newly-formed cytoplasmic vesicles generated by the expansion of goblet-like phages (or isolated membranes), which surround the cytoplasm and eventually seal the autophagosomes with a double-membrane structure (Figure 1). The origin of phagohore is still unclear. However, there were two notions that it originated from the endoplasmic reticulum (ER) or the membrane probably emerged from the fused cage-like tubular network [20]. The outer membrane of autophagosomes would fuse with tonoplast, followed by the release of inner vesicles termed as autophagic bodies. Regarding both microautophagy and macrophage, the disruption of the autophagic membrane releases the intraluminal components to the vacuole for hydrolyzing, and finally the cargo is digested into their constituents for the turning back to the cytoplasm. The most extreme form of autophagy is mega-autophagy, of which the vacuolar membranes were penetrated or ruptured to release vacuolar hydrolases directly into the cytoplasm, leading to the degradation of cytosolic materials [21]. Mega-autophagy initially symbolizes the final stage of programmed cell death (PCD), which occurrs during development or in response to pathogenic invasion [22].

In the past decades, autophagic machinery has emerged and been illustrated in accordance with the genetic screens. The responsible *ATG* genes, for macro- and microautophagy, pexophagy, chlorophagy and mitophagy etc., have been broadly identified based on the conserved homologues of ATGs between yeast and plant species, such as Arabidopsis and rice. These ATG proteins and other components demonstrated a canonical route for macroautophagy (hereafter referred to as autophagy) machinery. Among these factors, the ATG1 kinase and its regulatory protein ATG13 are phosphorylated by the TARGET OF RAPAMYCIN (TOR) and other kinases, and they then bind to scaffold protein ATG11 and associated protein ATG101 to form complexes and eventually initiate the autophagy in response to developmental and nutritional cues (Figure 1). The activated complex, of particular ATG1, promotes ATG9-mediated delivery of lipids to the emerging phagophore, leading to the expansion of membranes and vesicle nucleation accompanied by the interaction with ATG18 (Figure 1). In parallel, the decoration of the phagophore is also performed by the SRC HOMOLOGY-3 (SH3) domain-containing protein 2(SH3P2) and the conjugation of ATG8 to PE (ATG8-PE) adduct (Figure 1). During selective autophagies, ATG8s regularly interact with cargo receptors containing ATG8-interacting motifs (AIM), which consist of a consensus sequence (W/F/Y-X-X-V/I/L) that preferentially forms a negative charge (such as the presence of acidic acid of the X amino acid) and brings them into the autophagosome. In addition, a current research has identified a new type of ATG8-interacting protein containing the ubiquitin-interacting motif (UIM) [23], which would also be turned over as the same manner as that of AIM proteins. The sealed autophagosome is transported to the vacuole and then fused with the tonoplast with the help of FREE1 and other components (Figure 1). Finally, the autophagic body is released into the vacuole, in which the inner material of autophagosome is gradually degraded by vacuole hydrolases and the nutrients are recycled for the newly emerging tissue (or cell) use (Figure 1). Therefore, combined cell biology and genetic analyses of the relevant mutants involved in the above route enable us to explore extensively the exact regulatory mechanism(s) and manipulation of autophagy, as well as uncovering the novel regulators participating in the regulation of autophagy.

## 3. Regulation of Plant Autophagy

Plant autophagy broadly occurs in response to various stressful environments or at certain stages of growth and development. To ensure rapid adaptation to changing environments, the selective autophagy generally works in a timely and appropriate manner to maintain cellular homeostasis [24,25]. The utilization of chemical inhibitors and activators combined with genetic approaches allows us to dissect the molecular mechanisms underlining plant autophagy.

As mentioned above, the TOR is one of the most important factors in regulating the induction of autophagy. In the case of TOR inactivation, the expression levels of many *ATG* genes were significantly up-regulated in *Arabidopsis* roots, resulting in the activation of the autophagy system [26]. Overexpression of TOR inhibited autophagy induction upon nutrient starvations and salt and osmotic stresses rather than oxidative stress or ER stress [27,28]. Tap42/α4, a regulatory subunit of protein phosphatase 2A (PP2A), was known as a downstream effector of the TOR, of which the depletion reproduced similar trends to TOR inactivation, including the retardation of plant growth and the activation of autophagy [29]. Previously, the Arabidopsis Mei2-like 1 (AML1), ErbB-3 epidermal growth factor receptor binding protein (EBP1) and S6 kinase (S6K) have already been implicated to act downstream of TOR, and the defect or up-regulation of these proteins resulted in the multiple variations of plant growth and development [24,30,31]. However, little is known about their role in autophagy regulation. Therefore, these collections ultimately defined that the TOR kinase functions as a negative regulator of plant autophagy.

Referring to the positive regulation of autophagy, there were two known pathways mastered by the SH3P2 and the adenosine 5′-monophosphate kinase/Sucrose Non-fermenting 1-Related Protein Kinase 1 (AMPK/SnRK1), respectively. The SH3P2 was characterized as a novel membrane-associated protein for autophagosome biogenesis in Arabidopsis [32]. Through the cell biology analysis, the SH3P2-GFP was found to specifically participate in the assembly of the phagophore site/preautophagosome structure (PAS) upon autophagy induction and to associate with endoplasmic reticulum (ER), supporting the notion that the membrane source of autophagosome was at least partially derived from ER. Suppression of *SH3P2* resulted in a severe growth defect (such as the developmentally lethal) and significantly inhibited the formation of the autophagosome. In addition, SH3P2 interacts with phosphatidylinositol 3-phosphate (PtdIns3P) and ATG8, and acts as a downstream regulator of the phosphatidylinositol 3-kinase (PtdIns3K) complex [32], suggesting its function in the membrane expansion or maturation of autophagosome. Afterwards, FREE1 was identified as a SH3P2 binding protein and formed a complex together with SH3P2 and PI3K proteins to regulate the fusion of autophagosomes with vacuoles, eventually leading to the degradation of autophagosome in Arabidopsis [33]. Accumulation of autophagosome was revealed in the *free1* mutant, as well as the accumulation prevacuolar compartments/multivesicular bodies (PVCs/MVBs), implying an unknown regulatory mechanism essential for both endosomal sorting complexes required for transport (ESCRT) machinery and the autophagy process. Recently, other roles of SH3P3 have also been illustrated, namely that it interacted with dynamin-related protein 1A (DRP1A) and targeted it to cell plates, thus eventually affecting the conversion of the fused vesicles to tubular structures during cytokinesis [34], and functioned as a ubiquitin- and ESCRT-I-binding protein in clathrin-mediated endocytosis [35]. Given the close relationship between endocytosis and autophagy, it would be interesting to explore whether and how the clathrin-mediated endocytosis converges with autophagy, as well as the identification of conjugation factors.

Similar to its yeast and mammalian counterparts, SnRK1 is required for autophagy induction under a wide variety of stress conditions. Overexpression of the SnRK1 subunit *KIN10* in Arabidopsis promoted the autophagy induction and autophagosome formation by affecting the phosphorylation of ATG1, which led to the delays in leaf senescence and better tolerance to nutrient starvations, while down-regulation of *KIN10* suppressed the induction of autophagy upon multiple stressful treatments. Interestingly, the suppression of autophagy induction in *kin10* mutant can be removed by further inhibition of TOR [36,37], indicating that *SnRK1* functions upstream of *TOR* in regulating autophagy. 

Besides the regulators described above, certain chemical reagents monitoring autophagy have already been developed. Each of them affects various steps of autophagy, thus facilitating the characterization of novel components in regulating autophagy and even for the genetic screening by employing the chemical inhibitors or activators. Details of these compounds have been summarized and discussed by a recent extensive review [1], and thus will not be mentioned again in the present review.

To gain more insights into the regulation of plant autophagy, at least two recent studies have paid efforts to identify the corresponding transcription factors (TFs) that modulate the transcriptional expression of *ATGs* [38,39]. Promoters of four *ATG8* genes were used to screen TFs by yeast one-hybrid assay, and 225 TFs from 35 families were isolated and GO-enriched into developmental and environmental processes. The *TGACG (TGA) motif-binding protein 9* (*TGA9*) was experimentally confirmed as a representative, of which overexpression significantly up-regulated the expression of *ATG8* and other *ATG* genes, as well as the survival rate from starvation [38], demonstrating a positive role of *TGA9* in regulating autophagy. A genome scale analysis of TFs-associated *cis*-elements of *ATG* genes was also performed in wheat. Unfortunately, the exact TFs have not yet been identified, even the vesicle-associated membrane protein 727 (VAMP727) was suggested to play an important role in the interaction network with *TaATGs* [39]. Overall, these attempts offer a strategy for further understanding of autophagy regulation and the responsible TF candidates.

As is known, ATG8 interacts with cargo receptors during autophagy regulation. An investigation into such receptors may shed new light on the knowledge regarding selective autophagy. To this end, about 40 of these receptors have been identified and described using multiple approaches, providing the possible underlying mechanisms about breakdown of specific proteins and protein complexes, protein aggregates and degrade organelles, as well as even invading pathogens. These mechanisms are usually termed with “-phagy”. For example, autophagy-dependent degradation of a chloroplast is labeled as chlorophagy, possibly through the Rubisco-containing body (RCB) and Atg8-ineracting protein 1-plastid body (ATI1-PS) pathways [40]. The autophagy-mediated turnover of peroxisome, namely pexophagy, was postulated to be handled by the interaction of PEX1, PEX6 and PEX10 with ATG8 [41,42]. Additionally, the aggrephagy that executed by NEIGHBOR OF BRCA1 (NBR1) [19], proteaphagy that regulated by proteasome subunit RPN10 [43], and mitophagy that operated by ATG11 [44] have also been well-characterized and thoroughly reviewed recently [1]. However, it is still a relatively ambiguous scenario for such a specific autophagic mechanism due to few reported components. It probably could be further appreciated by genetic and biochemical screenings, such as mutagenesis and yeast two hybrid.

Numerous studies have evidenced that autophagy is involved in regulating plant resistance to biotic and abiotic stresses, including nutritional, drought, osmotic, salt, heat, and oxidative stresses and so on. Therefore, deciphering the exact regulatory role of autophagy in such biological processes would substantially extend our view of its regulation and unknown functions [45,46,47]. Accumulating studies have drawn a fairly clear picture of phytohormone metabolism, transport, perception and signaling transduction [48]. However, the relationship between autophagy and phytohormones, in particular the phytohormone-mediated stress response, still needs to be further explored until the recent discoveries are reported.

## 4. Crosstalk between Autophagy and Phytohormones

Phytohormones play a comprehensive role in the regulation of plant growth and development under favorable or unfavorable environments. Using *atg* mutants defective in autophagy route is a genetically regular approach to address the issue of the crosstalk between autophagy and phytohormones. As an efficient, rapid, and high-fidelity technique, genome-wide profiling of the autophagy-deficient mutants becomes one of the most convenient strategies to enable the elucidation of its in vivo connection to the phytohormones’ pathways in response to various stresses, particularly nutrient starvation [49]. As shown below, related progress is generally described in respect to each phytohormone with autophagy.

### 4.1. Crosstalk between Autophagy and Phytohormones upon Various Environmental Stimuli

Plants are sessile organisms frequently challenged by abiotic stresses, which sometimes result in accumulation of ABA. The Arabidopsis *Tryptophan-rich Sensory Protein* (*AtTSPO*), encoding a membrane-spanning protein responsible for osmotic and salt stresses, was induced by ABA and directly bound to ATG8 via its AIM-dependent interaction. Degradation of AtTSPO was attenuated in the autophagy-defective mutant *atg5* and was sensitive to the inhibitors of PI3K that were involved in regulating autophagy [50,51,52], suggesting a potential contribution of autophagy to the ABA-mediated stress response. In addition, two ATG8-interacting proteins, ATI1 and its homolog ATI2, binding ATG8 proteins via two putative AIMs, were also involved in the ABA-mediated germination in addition to salt tolerance [53,54]. Moreover, the *Heat-shock transcription factor A1a* (*HsfA1a*) TF can directly bind to the promoters of *ATGA10* and *ATG18f*, and then up-regulated the expression of these two genes and other *ATGs,* eventually activating autophagy in tomato (*Solanum lycopersicum*). However, it may be involved in ABA-mediated stomatal closure rather than the ABA-mediated drought response [55]. It is worthy to mention that mutation of Regulatory-Associated Protein of TOR 1B (RAPTOR1B) resulted in a strong reduction of TOR kinase activity, leading to induction of autophagy and elimination of ABA [56]. Further research revealed that TOR can also phosphorylate PYL ABA receptors and then inactivate the SnRK2 kinases under unstressed condition, while the ABA-activated SnRK2 can phosphorylate the RAPROR and then result in the dissociation and inhibition of the TOR complex under stress [57]. Considering the ABA responsive elements present in promoters of *ATGs* [39], it is proposed that the TOR pathway represses the ABA signaling under favorable conditions while the ABA signaling feedback regulates the induction of autophagy upon stress.

Numerous studies have further explored the crosstalk between autophagy and BR signaling under environmental stresses. The TOR signaling monitors the abundance of Brassinazole-resistant 1 (BZR1) to maintain the growth upon carbon starvation in Arabidopsis [58], implying a contribution of autophagy to the homeostasis of BR signaling. Subsequently, the BRI-EMS SUPPERSSOR 1(BES1), a transcription factor positively regulating BR signaling transduction, was also shown to be selectively turned over during drought and starvation stresses via the autophagy pathway mediated by the cargo receptor DOMINANT SUPPRESSOR OF KAR 2 (DSK2) [59]. Intriguingly, silencing of *BZR1* attenuated the transcript levels of *ATGs* and the formation of autophagosomes, which were enhanced in the *BZR1*-overexpressing plants after BR treatment. Knockdown of *ATG2* and *ATG6* compromised the formation of BR-induced autophagosomes. In addition, loss-of-function of BZR1 compromised the resistance to nitrogen (N) starvation but enhanced it in *BZR1*-overexpressing and exogenous BR-treated plants [14]. These results indicated that *BZR1* feedback regulated autophagy machinery by modulating the expression of *ATG2* and *ATG6* under nitrogen starvation in tomato.

Ethylene is also a stress response phytohormone like ABA. However, transcriptomic analysis found that only a few of ethylene (ET)-signaling associated genes, like *ETHYLENE RESPONSE2* (*ETR2*) and *CONSTITUTIVE TRIPLE RESPONSE1* (*CRT1*), were significantly induced in *atg5 atg9* double mutant [60]. Conversely, activated mitochondrial alternative oxidase (AOX) increased the level of autophagy whereas knockdown of *AOX1a* decreased the level of autophagy in ET-induced drought tolerance in tomato, which seems to be related with the changing level of reactive oxygen species (ROS). Moreover, it was shown that ET induced autophagy upon drought response through ERF5 binding to the promoters of *ATG8d* and *ATG18h* [60]. Nevertheless, the inhibition of autophagy by its inhibitor 3-methyladenine (3-MA) could be rescued by exogenous ET in banana (*Musa nana Lour*) [61]. Besides their crosstalk under stress, a previous study has been implicated in a possible involvement of ET in the autophagy-dependent pollen development, in which ET treatment obviously increased the expression of *ATG8* homologues in petals of petunia (*Petunia hybrida*), and the rising autophagosome formation during pollination was accompanied by increasing ET production [62]. In addition, a type of gametocide, acetolactate synthase (ALS)-inhibiting herbicides amidosulfuron (Hoestar), caused male sterility in parallel with the up-regulation of *ATG8A* and ET responsive TF *RAP2-11-like* in rapeseed (*Brassica napus L.*) [63]. AP2-EREBP (APETALA2/ethylene-responsive element binding proteins) might be the upstream regulator of autophagy since its responsive elements are present in the promoters of various *ATG* genes [38,39]. According to these findings, ET seems to interplay with autophagy in both plant development and tolerance.

It is well known that jasmonic acid (JA) is another phytohormone functioning in both biotic and abiotic stresses. It affected very few differentially expressed genes during plant senescence, such as *ETHYLENE RESPONSE FACTOR2* (*ERF2*) and *PLANT DEFENSIN 1.2* (*PDF1.2*), through the transcriptome profiling of senescing Arabidopsis *atg* mutants [60]. JA has been well documented to regulate *ERFs*, which function as an inducer of resistance to many abiotic stresses. The expression of JA signaling genes was dramatically altered during TOR inhibition [27,28,64]. Since the putative JA-related ERF element present in the promoter of *ATGs* [38,39], it would be a potential crosstalk between JA-related ERFs and TOR-mediated autophagy.

Salicylic acid (SA) metabolism and signaling have both been explored in regulation of autophagy. In general, autophagy deficiency has resulted in early leaf senescence and excessive immunity-related programmed cell death (PCD) in plants. In the past decades, accumulating evidence has indicated that the abnormally high level of SA but not JA or ET was the major result of autophagy-mediated senescence and PCD. Moreover, the application of SA chemical activator induced the senescence/PCD phenotype in SA-deficient *atg* mutants, but overexpressing the bacterial SA hydroxyplase protein *nahG* in *atg* background resulted in the stay-green phenotype [65]. However, the other phenotypes of *atg* mutants, like short hypocotyl, were not relative to SA [66]. Metabolomic and transcriptomic analyses revealed that both SA biosynthesis and accumulation up-regulated dramatically in *atg* mutants, especially under low nitrate conditions and carbon starvations [60,66]. Moreover, co-expression analyses of the transcriptome profiles of *pad4* and *npr1* with *atg* mutants clearly stated highly similar patterns among them, further suggesting that autophagy is involved in the SA-mediated stress responses [60]. Except for the abiotic stress response, autophagy and SA also function together in the biotic tolerance. For example, *MdATG18a* has a positive influence on drought tolerance [16], but also alters the resistance to *Diplocarpon mali* accompanied by enhancing SA abundance in apple [67]. A working model is proposed that SA accumulation leads to the burst of ROS, which in turn induces autophagy, while autophagy is able to reduce ROS production; thus, this provides a negative feedback-regulation mechanism [46]. Further studies are still expected to point out the selectivity and specificity of autophagic events during SA responses.

### 4.2. Autophagy Interplays with Phytohormones during Plant Development

Previously, it had been shown that the overexpression of the *AtATG8* gene had no obvious phenotypic variation in the root architecture as compared to that of wild-type (WT) plants under auxin treatments [68]. However, disruption of the autophagic system resulted in inhibition of both lateral root growth and auxin accumulation in the root tips under phosphate starvation [69]. Nevertheless, exogenous application of auxin, 1-naphthaleneacetic acid (NAA), arrested the accumulation of autophagic body by blocking autophagosome formation rather than degradation during nutrient deficiency, salt and osmotic stresses, but not during oxidative or ER stresses [27], which is consistent with the TOR-mediated autophagy regulation. Furthermore, enhanced activity of root meristem by glucose in the Arabidopsis *atg* mutants was detected to be associated with improved auxin gradients and auxin responses, indicating that autophagy is critical for regulating the levels of peroxisome and ROS, thus modulating the auxin activity [70]. These findings suggest that auxin-involved stress-induced autophagy depends on the TOR pathway. Notably, putative IAA- and ARF-elements were found in the promoters of *ATG* genes in wheat [38,39], implying an unknown transcriptional regulation of autophagy upon stress response. Therefore, isolation and characterization of relevant TFs might implicate the interaction of autophagy and auxin in response to stress.

Interestingly, *ATG8*-overexpressing plants exhibited less lateral roots and were hypersensitive to cytokinins (CKs) rather than WT as the shorter root length and less numbers of primary root [68], indicating an effect of autophagy on the CKs-dependent phenotype. Recently, a co-expression analysis of transcriptome profiles revealed that numerous genes that down-regulated in the cytokinin response deficient mutants, such as the *arr1 arr10 arr12* triple mutants, were up-regulated in the *atg* mutants, while a group of genes were both up-regulated in the cytokinin receptors defect mutant (*ahk2 ahk3 ahk4* triple mutants) and *atg* mutants, demonstrating that these genes might be the key factors linking autophagy and cytokinin [60]. An investigation into the endogenous level of phytohormones showed a decline in CKs in the anther of *osatg7-1* mutants [71], which perturbed male reproductive development [10,72]. In addition, the *MYB DOMAIN PROTEIN2* (*MYB2*) TF, known to regulate plant senescence by inhibiting CK-mediated branching [73], was significantly up-regulated in both *atg5* and *atg9* mutants under both low and high nitrate conditions. Taking into account the similar role(s) of autophagy and CKs in regulating N metabolism, it is speculated that autophagy participates in the CKs signal perception regardless of N starvation or other stresses. The lower level of gibberellin (GA) was also detected in the rice *osatg7-1* mutant as compared with WT [71], which was apparent as reduced plant height [10]. However, little is known about the relationship between them underlying the plant architecture. As shown previously, autophagy interplays with ABA in the seed germination [53]. Since GA antagonizes ABA in germination, it is reasonable to assume that GA also coordinates autophagy to regulate seed germination. REGULATORY-ASSOCIATED PROTEIN OF TOR 1B (RAPTOR1B) is an interacting partner of TOR, of which depletion (*raptor1b* mutants) is hypersensitive to extremely low concentrations of ABA, eventually leading to a significant decrease in the germination rate and significantly reduced root growth. Yet, these defects could be rescued with GA supplementation [74]. Therefore, when evaluating the coordination between autophagy and GA, it might be wise to focus on the seed germination in the future. 

**Figure 2:**
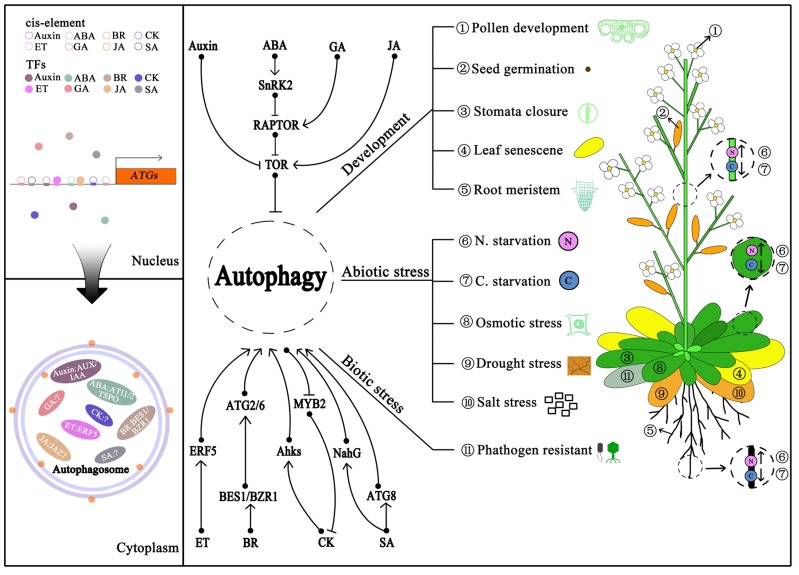
A proposed crosstalk between autophagy and phytohormones. The phytohormones-associated transcription factors (TFs) responsible for multiple biological processes are active or inactive during the plant growth, development and stress response. Upon certain conditions, these TFs are proposed to bind to the *ATG8* (and additional *ATGs*) promoter to trigger its expression, leading to the induction or inactivation of autophagy as indicated in the left upper square. Subsequently, the cargo receptors (such as phytohormone related proteins) and/or other biomolecules are delivered into the autophagosome, which is then transported into the vacuole for degradation as indicated in the left bottom squares. This process feedback modulates the homeostasis of phytohormones metabolism and signaling transduction at certain stages; eventually, this loop-regulation of each phytohormones with autophagy contributing to different aspects of the plant growth, development and stress response as representatively indicating in the right square from No.1 to No.11.

Taken all above together, these have provided important insights on the loop-regulation pattern underlying each phytohormone with autophagy for optimizing the balance of growth and stress responses in plants (Figure 2). Therefore, characterization of autophagy cargo receptors and the upstream or downstream TFs would indeed expand our understanding of the crosstalk between autophagy and phytohormones.

## 5. Contribution of Autophagy to Crop Productivity and Yield

Autophagy has broadly indicated its role in crop productivity and yield. For example, deletion of *OsATG7* caused the abnormal development of anther, sterile of spikelet, early leaf senescence, and low NUE, eventually leading to yield loss [10,13]. Further study revealed that anther defect of *osatg7* was partially due to the low endogenous level of bioactive GAs [71], which shed a light on the relationship between phytohormones and autophagy in terms of crop productivity. In addition, impaired maize *Atg12* also resulted in arrested seedling growth, accelerated leaf senescence and stunted ear development, as well as perturbed N remobilization, which significantly reduced the seed yield and harvest index [12]. Conversely, overexpressing *ATG5* or *ATG7* promoted growth, seed set and oil content in Arabidopsis [75]. Similarly, up-regulation of *ATG8* also remarkably increased the number of effective tillers in rice, and NUE in the grain of both rice and Arabidopsis, finally enhancing the yield without the penalty of plant development deficiency [15,17]. Collectively, these evidences clearly indicate that autophagy positively contributes to the crop productivity and yield, and the relevant *ATG* genes are great candidates for crop breeding and improvement.

Since autophagy participates in the regulation of phytohormones metabolism and signaling transduction as mentioned above, it is quite important and intriguing to further explore whether the autophagy-mediated crop productivity and yield is dependent or independent on certain phytohormone pathways. Nevertheless, most of the yield-related contributions of autophagy are associated with nutrients allocation and remobilization. However, little is known about whether the autophagy directly modulates other agronomic traits, including plant architecture, leaf morphology and grain shape in crop (such as rice and corn etc.). These issues are of great importance for the genetic manipulation of autophagy in crop breeding and improvement in the future.

## 6. Future Perspectives

Autophagy modulates plant development and stress resistance in line with phytohormone homeostasis, but there are still many open questions regarding the exact mechanism underlying the process regulation and specific interacting targets. ATG8 is the core component of autophagy machinery and binds receptors to operate the autophagic degradation [1,76]. In general, ATG8-interacting proteins generally contain certain AIMs. Therefore, high-throughput examining AIM containing proteins would be an alternative and convenient approach for identification of autophagy targets besides the conventional method, such as yeast two-hybrid screening. Two bioinformatics analyses, including iLIR and hfAIM, have been developed in an attempt to solve the issue [42,77]. The phytohormone-related proteins were also identified among the large-scale of potential targets by these analyses, providing specific candidates for understanding the autophagy-mediated homeostasis of each phytohormone during metabolic or signaling transduction processes. Nevertheless, a recent study has unraveled that a new type of UIM containing proteins can also interact with ATG8 [23], providing a wide range of candidates for further exploration of the phytohormones homeostasis with autophagy.

Additionally, most of the researches focus on the contribution of autophagy in multiple stresses, even crosstalk with phytohormones. However, it would be fascinating to ask if autophagy also participates in the phytohormones-mediated growth and development, in particular the context of agronomic traits, such as the productivity and yield, in addition to the NUE of crops. On the other hand, it is also attractive to see whether and how autophagy modulates the degradation of target proteins in competition with other systems, such as ubiquitin and hydrolysis, upon stressful conditions.

In summary, autophagy functions in biotic and abiotic stress responses to facilitate plant tolerance and survival from various unfavorable conditions. Due to the occurrence and severity of recent climate change, environmental pollution and world population growth, further exploring the regulatory mechanism of autophagy underlying the phytohormone’s pathway would further extend our knowledge, and then may provide new strategies and potential targets for the genetic improvement and breeding of new crop varieties for overcoming the challenge from environmental stresses.

## Abbreviations

ATG	Autophagy related gene
SA	Salicylic acid
JA	Jasmonic acid
CK	Cytokinin
ABA	Abscisic acid
ET	Ethylene
GA	Gibberellin
TOR	Target of rapamycin
